# Modulation of Voltage-Gating and Hysteresis of Lysenin Channels by Cu^2+^ Ions

**DOI:** 10.3390/ijms241612996

**Published:** 2023-08-20

**Authors:** Andrew Bogard, Pangaea W. Finn, Aviana R. Smith, Ilinca M. Flacau, Rose Whiting, Daniel Fologea

**Affiliations:** 1 Department of Physics, Boise State University, Boise, ID 83725, USA; 2 Biomolecular Sciences Graduate Program, State University, Boise, ID 83725, USA

**Keywords:** pore-forming toxins, lysenin, voltage gating, hysteresis, memory, bistability, Cu^2+^ ions

## Abstract

The intricate voltage regulation presented by lysenin channels reconstituted in artificial lipid membranes leads to a strong hysteresis in conductance, bistability, and memory. Prior investigations on lysenin channels indicate that the hysteresis is modulated by multivalent cations which are also capable of eliciting single-step conformational changes and transitions to stable closed or sub-conducting states. However, the influence on voltage regulation of Cu^2+^ ions, capable of completely closing the lysenin channels in a two-step process, was not sufficiently addressed. In this respect, we employed electrophysiology approaches to investigate the response of lysenin channels to variable voltage stimuli in the presence of small concentrations of Cu^2+^ ions. Our experimental results showed that the hysteretic behavior, recorded in response to variable voltage ramps, is accentuated in the presence of Cu^2+^ ions. Using simultaneous AC/DC stimulation, we were able to determine that Cu^2+^ prevents the reopening of channels previously closed by depolarizing potentials and the channels remain in the closed state even in the absence of a transmembrane voltage. In addition, we showed that Cu^2+^ addition reinstates the voltage gating and hysteretic behavior of lysenin channels reconstituted in neutral lipid membranes in which lysenin channels lose their voltage-regulating properties. In the presence of Cu^2+^ ions, lysenin not only regained the voltage gating but also behaved like a long-term molecular memory controlled by electrical potentials.

## 1. Introduction

Lysenin is a protein extracted from the coelomic fluid of the earthworm *E. fetida*, which inserts large β-barrel pores in artificial and natural lipid membranes [1,2,3,4,5,6]. The intricate, multi-step mechanism of pore formation implies binding to sphingomyelin (SM), oligomerization into prepores, and prepore-to-pore conversion [4,6,7,8,9]. The large diameter of the conducting pathway of the pore (~3 nm, [1,3,5]) leads to uncontrolled leakage of ions and molecules; this strong lytic activity dubbed lysenin as a pore-forming toxin (PFT) [5,7,8,9,10], although its physiological role is yet to be deciphered.

Reconstituted lysenin channels share features specific to ion channels such as large transport rate, regulation, and selectivity [2,10,11,12,13,14]. The intrinsic regulatory mechanisms manifest by adjustments of the channel’s conformation and conductance in response to stimuli of physical and chemical origin [13,14,15]. Lysenin channels reconstituted in artificial membrane systems comprising anionic lipids present voltage-induced gating at low positive voltages [2,16,17], but this feature vanishes when the channels are reconstituted in neutral membranes [2,11]. When exposed to multivalent cations, lysenin channels present ligand-induced gating in both charged and neutral lipid membranes [11,13,15].

A salient feature of lysenin channels is the prominent hysteresis in conductance and bistability, indicative of molecular memory [16,17,18,19]. Hysteresis manifests when the response to a particular stimulation is not fixed and depends on the history of the system [20]. All voltage-gated ion channels characterized by two states (i.e., open and closed) may present a dynamic hysteresis in conductance, resulting from the slow equilibration of the channels in response to periodic voltage stimuli [21]. However, other intrinsic mechanisms may come into play and endow ion channels with a more persistent hysteresis [20,22,23]. Any hysteretic behavior leads to bistability and acquisition of memory emerging from the history-dependent behavior [18,20,23], which paves the way for gaining novel functionalities originating in the non-Markovian distribution of the states explorable by the system under investigations. 

In the case of lysenin, the observed hysteresis is not dynamic, and seemingly originates in an invariant reopening pathway of the channels previously closed by applied positive voltages [16,18]. Notably, the reactivation pathway is also temperature-independent, while the inactivation pathway is strongly modulated by temperature variations [18]. This memristor-like behavior [18,24,25] manifests at time scales that greatly exceed the hysteresis observed for ion channels [26,27,28,29,30]. The hysteresis in conductance of lysenin does not vanish when the period of the oscillatory voltage stimulus is much larger than the relaxation time of the channels [18], which would be the hallmark of dynamic hysteresis [21]. 

The hysteretic behavior of lysenin channels is intimately linked to two important regulatory mechanisms, unique among PFTs, namely, voltage-induced gating and ligand-induced gating. To date, no reasonable attempt to advance a realistic model of lysenin regulation has been made, and this is justified by the multiple challenges accompanying such an attempt. A realistic model of gating must account for all regulatory features investigated so far regarding voltage gating, ligand gating, and hysteretic behavior. However, the regulatory response to physical and chemical factors is far from uniform. K^+^ ions and higher pH lead to a right shift in the voltage-induced gating, suggesting modulation of voltage regulation by electrostatic interactions [14]. The same monovalent cations adjust the hysteresis in conductance by shifting the voltage needed to close the channels to higher values, yet the reactivation pathway is rather invariant [16]. Multivalent ions present a more intricate interaction with lysenin channels, dominated by the resulting ligand-induced gating [11,13,15,31]. Many trivalent metal cations (lanthanides, Al^3+^, Fe^3+^) force the individual channels to adopt a fully closed state, while divalent metal cations (Ca^2+^, Mg^2+^, etc.) induce stable conformational changes characterized as sub-conducting states [11,13,15]. In most cases, the inhibitory effects are suppressed upon ligand removal by precipitation or chelation, indicative of reversibility. However, Cr^3+^ ions present a distinct inhibition pattern, suggesting cooperativity, and the changes are not reversible [15]. In the same line of intricacy, voluminous organic cations (spermidine, spermine) present an inhibition profile resembling divalent metal cations [13,15], suggesting that charge density as opposed to charge alone influences the pathway adopted for conformational changes (i.e., full closing, as opposed to sub-conducting). La^3+^ ions, at concentrations sufficiently small to render ligand-induced gating negligible, influence hysteresis similarly to monovalent cations: a right shift of the voltage gating and open probability during ascending voltage ramps, and a rather invariant reopening pathway during descending voltage ramps [16]. The interaction with anionic ATP adds another level of intricacy: the conductance is inhibited in a concentration-dependent manner, yet voltage-gating and open probability show a strong right shift for both ascending and descending voltage ramps [16,31]. 

These prior investigations are indicative of major hurdles in elaborating a realistic model of gating, yet they may provide clues required for a better understanding of regulatory mechanisms and hysteretic response. We attempted to explain gating and hysteretic behavior by considering electrostatic screening of a voltage-domain sensor moving into the hydrophobic core of the membrane [16], but no strong evidence for such a mechanism exists. Therefore, any additional clue on lysenin’s functionality modulation by physical or chemical cues may contribute to the development of a realistic model of regulation. To gain new knowledge on the functionality of lysenin channels, we employed electrophysiology approaches to assess the effect of Cu^2+^ ions on the voltage regulation and hysteretic behavior. Unlike other multivalent metals that force the channels to close in a single step or to adopt a stable, sub-conducting state [11], Cu^2+^ ions interact with lysenin and lead to full closing in two steps [11,13]. To verify if this distinct interaction between lysenin channels and Cu^2+^ ions influences the hysteresis in conductance, we employed electrophysiology measurements and concluded that the addition of small amounts of Cu^2+^ ions not only significantly enhances the hysteretic behavior but also enables its persistent manifestation at zero bias voltage. This result prompted us to further investigate the effect of Cu^2+^ ions on the voltage-gating of lysenin channels reconstituted in neutral membranes, for which such feature is abrogated [2,11,15]. Surprisingly, Cu^2+^ addition led to a full restoration of the voltage-induced gating of lysenin channels reconstituted in neutral membranes, which also manifested a strong hysteresis in conductance.

## 2. Results and Discussion

**Figure 1:**
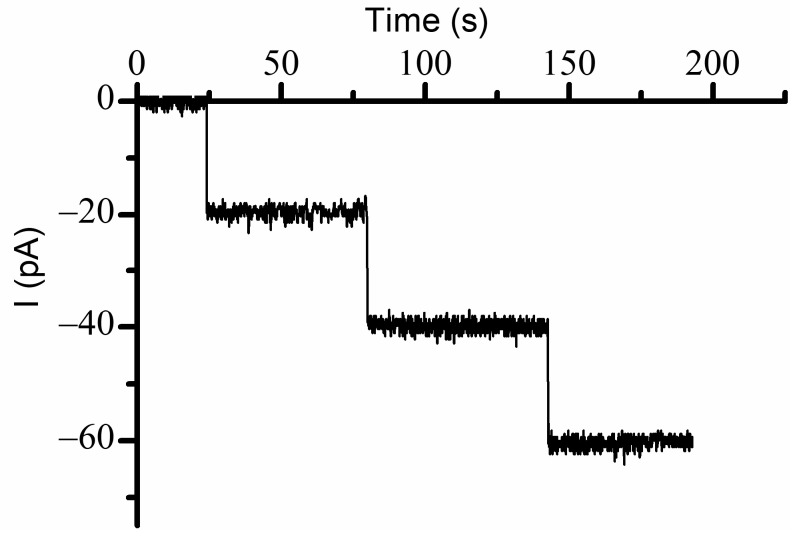
Lysenin inserts uniform channels in artificial lipid membranes. The insertion of individual lysenin channels in a planar bilayer lipid membrane was monitored from the stepwise variation of the ionic currents at −60 mV transmembrane voltage. Each inserted channel adjusted the ionic current by ~20 pA.

Our experiments were initiated by reconstituting lysenin channels in a planar Bilayer Lipid Membrane (BLM) composed of Asolectin (Aso), Sphingomyelin (SM), and Cholesterol (Chol), bathed by electrolyte solutions (50 mM KCl, 20 mM Hepes, pH 7.2). We opted for a low electrolyte concentration for several reasons: lysenin channels gate at lower voltages [14,16], a low conductivity solution enables measuring ionic currents through very large populations of reconstituted channels [19], and a lower ionic strength may enhance the electrostatic interactions by reducing screening. Channel insertion was monitored at −60 mV bias potential, and the insertion of individual channels was inferred from the stepwise variation of the ionic currents (Figure 1). Each inserted channel led to a variation of the ionic current by ~20 pA, consistent with previous experiments carried out in similar solution and electrical conditions [19]; the resulting conductance (~0.33 nS/channel) was used to provide a rough yet realistic estimation of the number of reconstituted channels for each of the experiments from the slope of the linear portion of the I-V plots [13,19] or the macroscopic currents measured at a voltage at which all the channels are fully conducting. 

**Figure 2:**
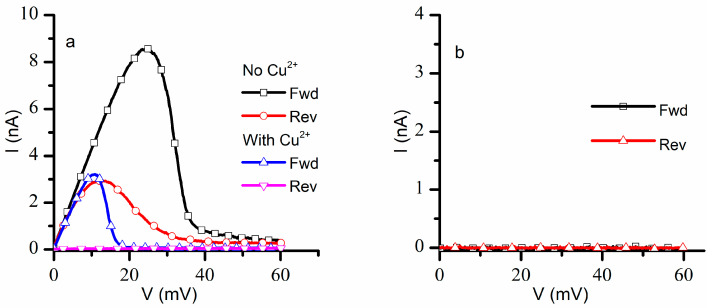
Cu^2+^ ions adjust the voltage gating and hysteresis in the positive voltage range. (**a**) The I-V plots recorded for forward and reverse voltage ramps before and after Cu^2+^ addition indicate major adjustments in channel closing and reopening. Cu^2+^ addition reduces the voltage required to initiate gating during ascending (Fwd) voltage ramps and elicit resistance to reopening during descending voltage ramps (Rev). (**b**) The negligible ionic currents recorded for a consecutive voltage ramp applied to Cu^2+^ -exposed channels indicates the persistency of the closed state of the channels. The panels show experimental data from single traces, with the symbols added to facilitate identification.

The next investigations focused on recording the response of a population of ~1300 lysenin channels to a slow oscillatory voltage stimulus in the positive voltage range with and without addition of Cu^2+^ ions. For this task, we measured the ionic currents in response to linearly variable voltage ramps (ascending and descending) ranging from 0 mV to +60 mV with a period of 20 min at a sampling rate of 1 Hz, a protocol customarily used to assess the hysteretic behavior of lysenin’s conductance [16]. In the absence of Cu^2+^ ions (control experiment), the lysenin channels showed the typical hysteretic behavior [16,18] (Figure 2a). During the ascending voltage ramp, the currents increased linearly with voltage until ~20 mV, after which the channels started to close, and the ionic currents became negligible at voltages greater than 45 mV. The closed channels started reopening at lower voltages during the descending voltage ramp, and full reopening occurred at voltages under 10 mV. Given the known influence presented by monovalent and trivalent metal cations on the hysteretic conductance of lysenin channels [16], we expected Cu^2+^ ions to elicit a right shift in the voltage gating during the ascending voltage ramp, and an invariant reopening pathway during the descending voltage ramp. However, the I-V plot recorded in response to otherwise identical voltage ramps after Cu^2+^ addition was quite different (Figure 2a): the channels started closing at a much lower voltage (<10 mV) during the ascending voltage ramp, and minimal reopening occurred during the descending voltage ramp. The hysteresis in conductance persisted but the channels resisted reopening after Cu^2+^ addition. 

A consistent overlap of the I-V plots was observed for three consecutive runs within the same experiment in the absence of Cu^2+^ ions. However, the I-V plots recorded after Cu^2+^ addition changed significantly following the first run: the closed channels did not reopen after the first run, and only negligible ionic currents were recorded for both ascending and descending voltage ramps (Figure 2b). The attempt to record data from independent experiments proved futile, owing to difficulties to replicate the exact same number of inserted lysenin channels in independent experiments. The insertion process is not easily controlled, and the number of inserted channels may differ substantially between experiments carried out in otherwise identical experimental conditions, which would make a statistical analysis of I-V plots meaningless. One may argue that a normalized value, like the open probability, would be suitable for statistical analyses of data from independent experiments; this is not necessarily true, since it was shown that the open probability may depend on the number of inserted channels [19]. Consequently, our results represent typical, single traces recorded in individual experiments. However, each set of paired data showing the effects of Cu^2+^ on a particular feature comprised the same membrane, with measurements taken before and after Cu^2+^ addition. This limitation is not uncommon: single traces are reported for similar electrophysiology experiments in which the number of channels cannot be precisely controlled [27,28,29,32], including plots of normalized quantities. 

**Figure 3:**
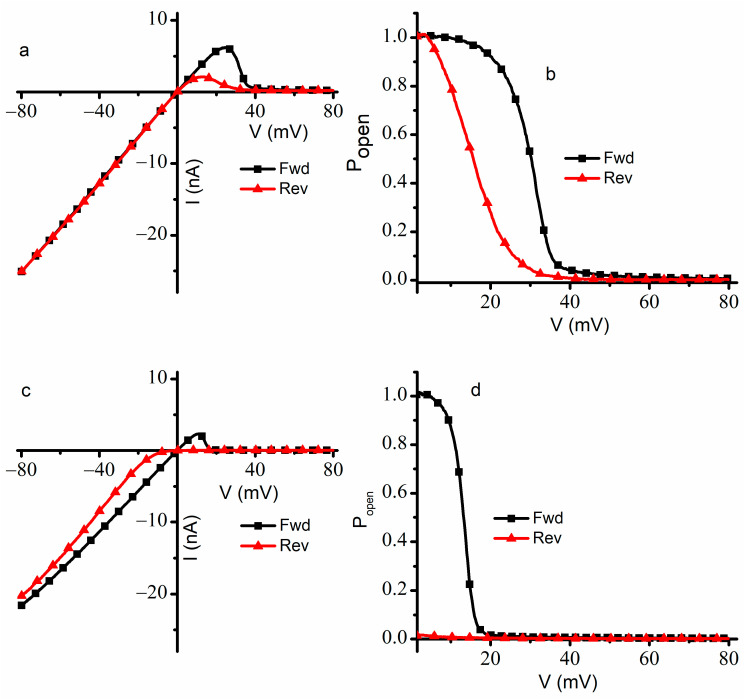
Cu^2+^ ions modulate the voltage gating and hysteresis of lysenin channels for an extended voltage range. In the absence of Cu^2+^ ions, the hysteresis in conductance in response to ascending and descending voltage ramps is observed for I-V (**a**) and open probability (P_open_) (**b**) plots. The changes in the macroscopic currents, P_open_, and midway voltage of activation for channels in a previously open state indicate a history-dependent response to applied voltages. Cu^2+^ addition influences the I-V (**c**) and P_open_ (**d**) plots recorded in response to the oscillatory voltage stimuli. The addition of Cu^2+^ ions induces a strong leftward shift in gating during ascending voltage ramps and the previously closed channels resist reopening. The traces represent experimental data, from single traces, with the symbols added to facilitate identification.

For a better understanding of the effects presented by Cu^2+^ ions on gating, we repeated the experiments by extending the voltage range from −80 mV to +80 mV and the ramp period to 30 min (Figure 3) for a population of ~950 channels. In the absence of Cu^2+^ ions, the I-V plot recorded in response to the ascending voltage ramp showed the typical response of lysenin channels to external voltages [14,16,18,19]. From −80 mV to +20 mV, the ohmic relationship between macroscopic currents and voltages indicated that the channels remained in the open state (Figure 3a). The channels began to close at voltages exceeding +20 mV; this behavior continued up to the maximum applied voltage of +80 mV. The steep decrease in the macroscopic currents at voltages over +30 mV indicated voltage gating and sustained channel closure; the effectiveness of voltage gating at positive potentials was inferred from the very small ionic currents recorded at voltages greater than +50 mV. After nearly all the channels were closed by the large depolarizing voltage, the application of a descending voltage (from +80 mV to −80 mV) led to the observation of hysteresis in conductance (Figure 3a). The reopening of the lysenin channels followed a different pathway: the channels showed a preference for the closed state for a larger voltage range and fully reopened at lower positive voltages. At descending voltages under ~+8 mV, the linear I-V plot indicated an ohmic behavior identical to the I-V plot recorded for ascending voltages, confirming a complete reopening of the channels.

The observations inferred from the I-V curves recorded in the absence of Cu^2+^ ions were confirmed by plots of the open probability (P_open_) determined in the positive voltage range for ascending and descending ramps (Figure 3b). To calculate the P_open_ value, we used the ratio between the measured currents and the theoretical maximal currents estimated for the same population open channels assumed in the open state at each applied voltage [19,33,34]. These theoretical currents were approximated from the slopes of the linear portion of the response to ascending voltages [18,19]. Since a P_open_ value of one was obvious at negative voltages and to avoid the division by zero around the origin, the P_open_ was plotted only for positive voltages larger than 1.5 mV. During the ascending voltage ramp, the P_open_ equaled one at low positive voltages, started decreasing as the voltage increased, and approached zero at transmembrane voltages larger than 50 mV. However, for the descending voltage ramp, the plot was shifted to the left, and a full reopening occurred at voltages of a few mV. The midway voltage of activation V_0.5_ (i.e., the voltage at which P_open_ = 0.5) [19,35,36] for ascending ramps was estimated at ~32 mV, while the descending ramps indicated a smaller V_0.5_ value of ~15 mV, which is consistent with earlier reports on the hysteresis and bistability of lysenin channels subjected to slow oscillatory voltages [18].

Substantial qualitative and quantitative differences were observed when the experiment was repeated in the presence of 4 µM Cu^2+^ ions added to both sides of the membrane (Figure 3c). As a consequence of the ligand-induced gating presented by the interactions between lysenin channels and Cu^2+^ ions [11], the recorded ionic currents were lower for the entire voltage range. The I-V plot recorded during ascending voltage ramps was linear from −80 mV to ~+8 mV, which is indicative of an ohmic behavior and the absence of voltage-induced gating within this range. We concluded that the Cu^2+^ addition adjusted the voltage at which the channels started to close, and gating occurred at much smaller voltages than in the absence of Cu^2+^ ions. This is not the typical behavior of lysenin channels exposed to monovalent and multivalent metal cations, for which the addition induces a rightward shift in the voltage gating behavior during ascending voltages [16]. The channels practically closed at +20 mV and remained in this state for applied voltages up to +80 mV.

A peculiar behavior regarding the effects of added Cu^2+^ ions was observed during the descending voltage ramps. The ionic currents measured from +80 mV to 0 mV were very small, indicating that lysenin channels resisted opening at any positive voltage. This is also contrasted with the typical behavior of lysenin channels exposed to multivalent metal cations, in which case the reactivation pathway, although indicative of hysteresis, is invariant and unaffected by ion additions [16]. The addition of Cu^2+^ ions not only elicited an early closing during ascending voltage ramps but also forced the voltage-closed channels to stay in that state for the entire range of positive potentials during descending voltage ramps. In addition, it seems like the channels remained closed even at negative voltages, and sustained reopening began at voltages under −10 mV. The nonlinear shape of the descending I-V plot recorded at negative voltages together with the consistently lower values of the ionic currents suggested that the reopening of the channels continued at negative potentials and that not all the channels reopened within the timeframe of the experiment. Since the channels did not fully reopen during the descending voltage ramp, we used the linear portion of the I-V plot recorded during the ascending voltage ramp as reference to determine P_open_ since all the channels, except the ones closed due to ligand gating, were in the open state at negative voltages. The analysis of the P_open_ at positive voltages (Figure 3d) confirmed our observations inferred from the I-V plots. The midway voltage of activation V_0.5_ for the ascending voltage ramp was estimated at ~+15 mV; however, the P_open_ measured for the descending voltage ramp was nearly zero for the entire range of positive voltages.

**Figure 4:**
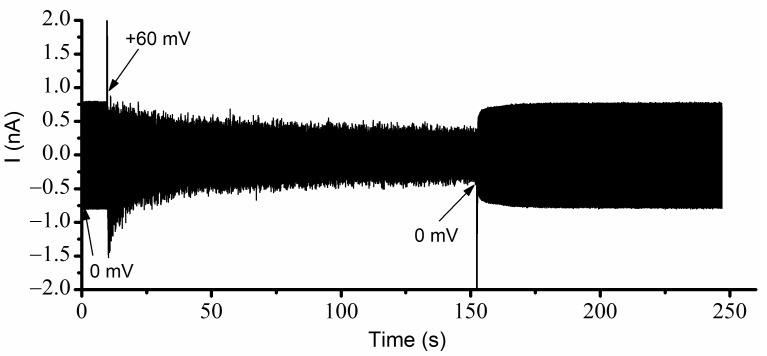
Determination of lysenin channels’ macroscopic conductance from combined AC/DC stimulation. The application of 0 mV and +60 mV DC voltages is indicated in the figure. The fully open state at 0 mV at the beginning of the recording is indicated by the large value of the AC current amplitude. The decreasing amplitude observed after the application of +60 mV indicates channel closure. The re-application of +60 mV reinstates the fully conducting state, which is indicative of channel reopening.

The experimental investigations of the effects of Cu^2+^ ions presented above showed major changes in the hysteresis and bistability of lysenin channels, indicative of memory capabilities. Nonetheless, an important remaining question was the behavior of lysenin channels in the absence of any applied voltage (i.e., 0 mV). The I-V and P_open_ plots for ascending ramps suggested a full opening when 0 mV was applied to channels that had previously been in an open state. However, for descending voltage ramps, the plots suggested that at 0 mV, the channels would remain in the closed state if their prior state was closed. To test this assumption, we employed the AC/DC setup presented in the Section 3 and determined the status of the channels regarding various voltages, prior electrical conditions, and conducting states. The resulting recording, taken of a large population of channels without Cu^2+^ ions and exposed to simultaneous AC/DC excitation, is shown in Figure 4 for each voltage condition. Before starting the experiment, the channels were biased for a few minutes at −60 mV to ensure that all channels were in the open state. The recording started immediately upon the application of 0 mV DC. In these conditions, the amplitude of the AC current through the ~2400 open channels was ~790 pA (Figure 3). The manual application of a +60 mV step voltage gradually reduced the amplitude of the AC signal down to ~375 pA, indicating a reduction in the macroscopic conductance due to channel closing induced by the applied voltage. After a few minutes, the reapplication of 0 mV led to channel reopening, which was observed as a quick increase in the amplitude of the AC current. In less than one minute, the amplitude of the AC signal recovered the initial value (~790 pA), indicative of a quick reinstatement of the original conductance and full channel reopening. This experiment suggested that, in the absence of Cu^2+^ ions, the channels were open at 0 mV, started closing at positive voltages, and rapidly re-instated their initial conductance after removal of the DC voltage stimulus.

A few important observations can be made from a further analysis of the channel conductance estimated from the trace shown in Figure 4. Firstly, the signal to noise ratio recorded at 0 mV before and after channel closure seemed much smaller than the one recorded during the application of the +60 mV step voltage. The larger value of the electrical noise while channels were closing most likely originated in the conformational open-close fluctuations manifested during voltage application. At +60 mV, the amplitude of the AC current decreased exponentially (as anticipated) but owing to the large volume of data (sampling frequency 500 Hz), we opted not to wait until a constant amplitude was achieved (i.e., indicative of steady state). Regardless, one may reasonably estimate that many of the channels were still in the open conformation, which was not observed in the I-V and P_open_ plots shown in Figure 2 and Figure 3. The explanation for this behavior is related to the high density of the channels in the target membrane [19]. To successfully complete these experiments and obtain a reasonable amplitude of the AC current for a 1 mV stimulation, we needed to reconstitute more channels in the target membrane. However, given the propensity of lysenin to prefer oligomerization into lipid rafts [19,37], high local channel densities may be achieved with only a few thousand channels, which may lead to adjustments of voltage-induced gating and the prevention of full closing even at high positive voltages [19].

**Figure 5:**
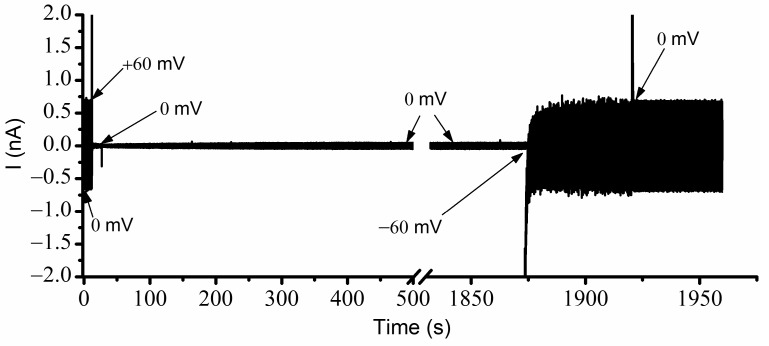
Cu^2+^ ions adjust the response of lysenin channels to voltages in a history-dependent manner. A large macroscopic conductance at 0 mV for previously open channels is indicated by the large amplitude of the AC current. The channels close rapidly at +60 mV but removal of the DC bias voltage does not lead to reopening. The channels reopen upon application of a negative bias voltage step (−60 mV); subsequent removal of the DC stimulus (i.e., reapplication of 0 mV) reinstates the current prior to channel closure, demonstrating a bistable system.

Our next experiment addressed the influence of Cu^2+^ ions on lysenin channel’s opening, reopening, and bistability in response to applied voltages while accounting for the history of the channels. To do this, we utilized the same experimental system and used the AC currents to determine the status of the channels exposed to 4 µM Cu^2+^ and additional DC voltages (Figure 5). Before recording, the membrane was biased by −60 mV for several minutes to ensure that all the channels were in the open state. The application of the AC signal at 0 mV DC voltage indicated an ionic current amplitude of ~710 pA. The application of +60 mV DC to the membrane led to a fast decline of the AC current amplitude to ~35 pA. After channel closure, we monitored the amplitude of the AC current at 0 mV bias voltage. As the experiment utilized a fast-sampling rate, we recorded a large amount of data. To mitigate potential problems with the acquisition system and further data analysis, we temporarily paused the recording during the experiment while maintaining all other electrical and solution conditions. As Figure 5 shows, a very small number of the lysenin channels previously closed by the application of the +60 mV potential reopened after removing the voltage stimulus. At 0 mV, the previously closed channels maintained their state for an extended time, as seen by the notably low AC current amplitude, which was monitored for more than 30 min. The small, stable amplitude of the AC current suggested the channels exposed to Cu^2+^ ions and previously closed by voltage remained in the closed state for a remarkably long time, if not indefinitely, at a 0 mV bias potential.

**Figure 6:**
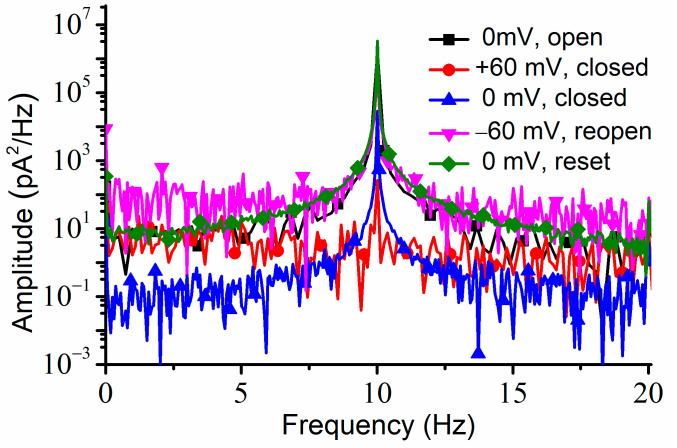
The power spectrum recorded for all the voltage and conformation conditions indicate the presence of the 10 Hz AC signal for channels open at 0 mV (squares), closed at +60 mV (circles), closed at 0 mV (up triangles), reopen at −60 mV (down triangles), and again at 0 mV after reopening by the negative step voltage (diamonds). The symbols were added to traces constructed from all the experimental data to facilitate identification.

To verify if the channels were permanently closed (which would be in contradiction with the results shown in the I-V plots), we applied a −60 mV transmembrane voltage for about one minute, which elicited a fast increase in the AC currents, which was indicative of channel reopening. The same maximum amplitude of the AC current was recorded after a consequent application of 0 mV; the recovery of the amplitude of the AC current to the same value we recorded at 0 mV before closing the channels suggested that the channels fully reopened upon the application of the hyperpolarization voltage. The power spectrum for each portion of the trace recorded at a particular voltage and channel conformation indicated the presence of the 10 Hz AC signal (Figure 6).

**Figure 7:**
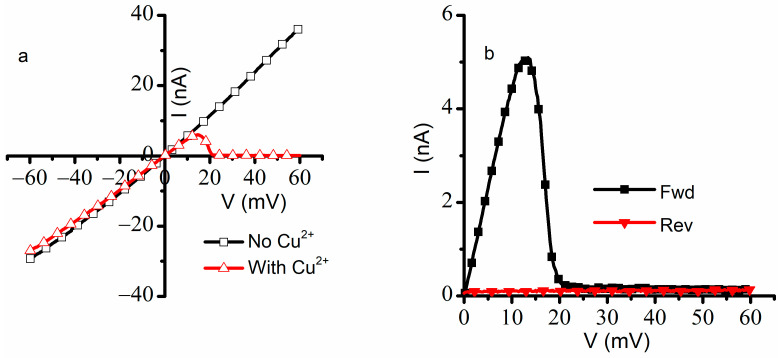
Cu^2+^ reinstates the voltage gating and hysteresis features of lysenin channels in neutral membranes. The linear I-V plot recorded for lysenin channels reconstituted in neutral membranes ((**a**), open squares) indicates the absence of voltage-induced gating. The voltage-induced gating feature is reinstated upon Cu^2+^ addition ((**a**), open up triangles). The I-V plot recorded after Cu^2+^ addition (**b**) for ascending (full squares) and descending (full down triangles) voltage ramps indicates the hysteresis in conductance and history-dependent response to applied voltages. The plots represent experimental data from single traces, with the symbols added to facilitate identification.

The experiments indicated that Cu^2+^ ions elicit not only major changes in hysteresis but also adjustments of the voltage-elicited response: faster closing, slower reopening, and a left-shifted midway voltage of activation. This is opposite to what is known of the influence of monovalent and trivalent metal cations on lysenin channels, which usually manifests by a rightward shift of the midway voltage of activation and slow down the response to positive voltage stimuli in response to ascending voltage ramps [14,16]. These discrepancies suggest Cu^2+^ may present a very different interaction with lysenin channels and act by promoting channel closure even when this feature would be weakened or suppressed by experimental conditions. In this line, lysenin channels are well known for losing their voltage-induced gating upon reconstitution in bilayer lipid membranes composed of electrically neutral lipids [2,11,15]. To investigate the influence of Cu^2+^ ions on the voltage gating of lysenin channels reconstituted in neutral bilayers, we employed support lipid membranes in which we replaced the anionic Aso with the neutral Diphytanoil-Phosphatidylcholine (DiPhyt-PC) as the major membrane component [2,11,15]. In these experimental conditions, the suppression of voltage gating for the ~2000 inserted channels was seen in the quasi-linear I-V plot recorded from −60 mV to +60 mV (Figure 7).

The linear I-V plot constructed for lysenin channels reconstituted in neutral membranes and in the absence of Cu^2+^ ions indicated that no voltage-induced gating manifested for the entire voltage range employed in this experiment, confirming prior investigations [11,15]. After the addition of Cu^2+^ to both sides (4 μM final concentration in each reservoir), the lysenin channels maintained the ohmic behavior for negative voltages and up to ~+10 mV. Surprisingly, the ionic currents decreased significantly for larger positive voltages (exceeding +10 mV), indicative of channel closing by voltage gating. The small values of the ionic currents measured at applied voltages exceeding 20 mV implied that the channels closed completely, suggesting that the Cu^2+^ addition restored the voltage gating properties. In addition, the P_open_ determined for voltage ramps in the positive voltage range (Figure 7b) indicated a complete recovery of the hysteretic behavior, including the arresting of the channels in the closed state at positive voltages and the absence of reopening at 0 mV.

Our findings raise more questions than provide answers with regard to deciphering the complex gating mechanisms presented by lysenin channels. Voltage gating and its absence in neutral membranes, as well as hysteresis, have been reported for more than two decades [2,18]. Nonetheless, no real progress was encountered regarding the biophysical mechanisms by which they manifest, which makes a mechanistic interpretation of our data difficult. Although electrostatic interactions seem to play a major role in voltage gating (because it is suppressed in neutral membranes, and modulated by ionic strength), how exactly they lead to gating is not known. Our work did not bring any experimental evidence to indicate that the changes in voltage gating and hysteresis are a consequence of the interaction of Cu^2+^ ions with lipids, proteins, or both. Cu^2+^ ions lead to a leftward shift of the open probability for both ascending and descending voltage ramps, while other monovalent and multivalent metal cations (i.e., K^+^ and La^3+^) adjust the hysteresis by a rightward shift of the open probability during ascending voltage ramps and an invariant pathway for descending voltage ramps (channel reopening) [16]. The current structural data [1,3] do not provide sufficient evidence of an outside domain able to occlude the channel by a ball and chain mechanism, but such occurrence may not be fully excluded. While our findings may help in elucidating the mechanism of lysenin’s gating, substantial investigations are needed to attain this goal. 

On the other hand, we anticipate our findings to contribute to a better understanding of how such behavior may provide molecular memory capabilities to unicellular or even complex organisms. Lysenin is not an ion channel; as a matter of fact, it is not even a transmembrane protein in its native environment. Lysenin shares salient features of ion channels (i.e., high transport rate, regulation, and selectivity), and it is endowed with strong memristive capabilities. The memory originating in the kinetics of ion channels as well as the implications of memristive properties in learning and the establishment of important neural functions are under intense scrutiny for a better understanding of how molecular memory contributes to fundamental physiological processes [20,22,23,32,38,39,40,41,42,43]. In addition to replicating fundamental features of ion channels, lysenin is much easier to work with, which makes it an excellent experimental model for exploring molecular memory phenomena and their consequences. 

## 3. Materials and Methods

The lipids used for these experiments were Asolectin (Aso, Sigma-Aldrich, St. Louis, MO, USA), Sphingomyelin (SM, Avanti Polar Lipids, Alabaster, AL, USA), Diphytanoil-Phosphatidylcholine (Diphyt-PC, Avanti Polar Lipids) and cholesterol (Chol, Sigma-Aldrich). The lipids, which were originally in powder form, were solubilized in n-decane (Fisher Scientific, Pittsburgh, PA, USA) to produce lipid mixtures of Aso/SM/Chol at a weight ratio of 10:5:4. Neutral bilayers were prepared by replacing Aso with DiPhyt-PC in the lipid mixture.

**Figure 8:**
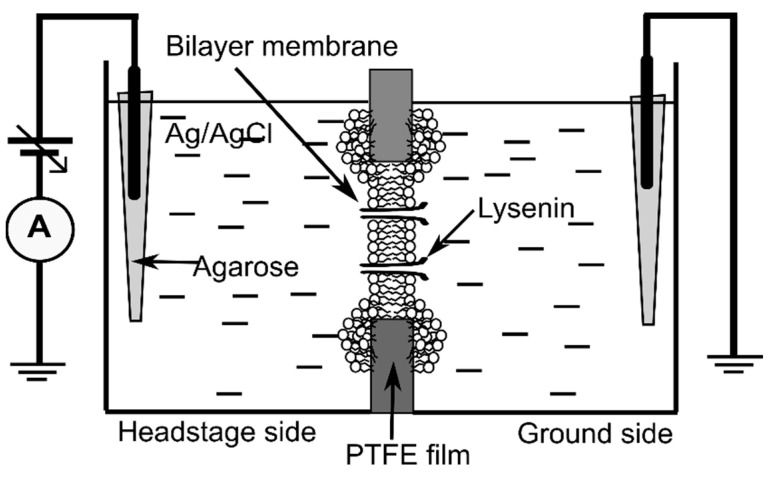
The experimental setup for electrophysiology measurements. Lysenin channels are reconstituted in a bilayer lipid membrane (BLM) bathed by electrolyte solutions. The electrical connections to the Axopatch 200B electrophysiology amplifier are ensured by agarose salt bridges and Ag/AgCl electrodes wired to the headstage. The diagram is not to scale.

The planar lipid membrane experimental setup (Figure 8), often used for electrophysiology investigations on lysenin channels [2,10,11,12], consisted of two insulating polytetrafluoroethylene (PTFE) reservoirs (each of ~1 mL volume) separated by a thin PTFE film (~120 µm thickness) in which a central hole of ~100 µm diameter was created by an electric spark. The reservoirs were filled with electrolyte solutions made of 50 mM KCl (Fisher Scientific) and 20 mM Hepes (pH 7.2, Sigma-Aldrich). For electrical connections, two Ag/AgCl electrodes embedded in salt bridges (2% low melting point agarose-Sigma Aldrich, dissolved in 1 M NaCl—Fisher Scientific) were inserted directly into the electrolyte solutions in the two reservoirs. The Ag/AgCl electrodes were wired to the headstage of the electrophysiology amplifier (Axopatch 200B, Molecular Devices, San Jose, CA, USA), which fed into the DigiData 1440A digitizer (Molecular Devices). The computer-controlled digitizer was used for online visualization, recording, and further analysis with the pClamp 10.7.0.3 software package (Molecular Devices). The recorded data were further analyzed and plotted with the Origin 8.5.1 (OriginLab, Northampton, MA, USA) software package.

Membrane formation and stabilization was monitored by estimating the membrane capacitance from the capacitive current measured in response to a triangle-wave signal (provided from a Keithley 3390 function generator), and the seal was verified by applying a DC voltage to the membrane. After a stable membrane was formed (C > 65 pF, R > 100 GΩ), we proceeded with channel insertion. Small amounts of recombinant lysenin [12] were added to the grounded reservoir under continuous stirring (Warner Instruments low noise magnetic stirrer) and upon application of −60 mV bias potential (manual command). Channel insertion was recorded at 10 Hz sampling frequency and monitored from the stepwise variation of the ionic currents measured at constant voltage [2,10,13,14]. After achieving a steady state of the macroscopic current in ~2 h, we proceeded with electrophysiology measurements before and after Cu^2+^ additions. For this purpose, we used a 1 M CuSO_4_ stock solution (Fisher Scientific) after proper serial dilution in buffered electrolyte solutions. After Cu^2+^ addition to both sides of the membrane (4 µM final concentration for all experiments), we maintained stirring for several minutes to allow mixing and enable interactions between channels and ions. 

Linear voltage ramps were created with the digitizer by defining proper episodic stimulation protocols [14,18]. For data recording, we used a 1 kHz low-pass hardware filter, 100 Hz low-pass software filter, and a sampling frequency of 1 Hz.

**Figure 9:**
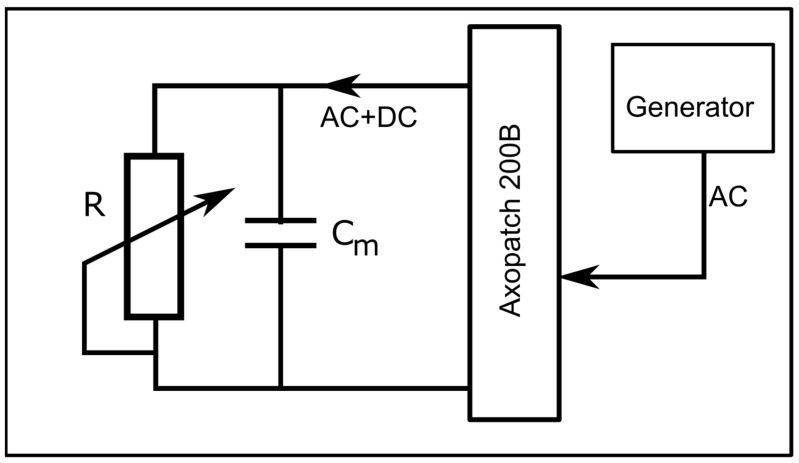
Experimental setup for investigating the status of the channels at any applied DC voltage. A combined AC/DC signal is applied from the electrophysiology amplifier to the membrane depicted as a capacitor C_m_ in parallel to a variable resistor R (the conducting pathway created by inserted lysenin channels).

To determine the status of the channels (i.e., open/closed) at any voltage (including zero voltage), we created an experimental setup that applied simultaneous AC and DC to the membrane. The DC voltage, utilized to control the status of the channels, was applied to the bilayer membrane with the manual command of the instrument, while the AC signals (10 Hz, 1 mV amplitude sinewave), utilized to estimate the status of the channels, were applied from the function generator via the external input of the electrophysiology amplifier (Figure 9). The unfiltered current included both AC and DC components, with each one dependent on the conductance of the channel population. The application of a high-pass 2 Hz filter suppressed the DC current component; the amplitude of the resulting AC current component was used to estimate the conductance status of the channels at any applied DC voltage.

The amplitude of the AC signal was chosen to be very small (i.e., 1 mV) to prevent channel closing by voltage (which may not occur at very low positive voltages, or fast sweeps [18]), yet it was large enough to detect AC currents through open channels. Also, the low amplitude and frequency prevented attaining large values of the capacitive currents; for a 100 pF membrane, the amplitude of the capacitive current estimated for these experimental conditions would be less than 10 pA. To avoid excessive chopping or filtering of the AC signal, we utilized a sampling time of 2 msec, a 10 kHz low-pass hardware filter, and no other low-pass software filter.

## 4. Conclusions

Our work showed that the addition of very small amounts of Cu^2+^ ions to the bulk electrolyte solutions had multiple effects on the voltage gating profile of lysenin channels. Earlier reports showed that lysenin channels reconstituted into lipid membranes containing anionic lipids present a strong hysteresis in conductance manifested as a preference for the previously attained closed state. This hysteresis did not originate in the slow equilibration of the channels since it persisted for periods of the oscillatory stimulus that greatly exceeded the relaxation time of the channels. In this work, we utilized traditional I-V measurements together with an improved experimental setup and showed that Cu^2+^ addition modifies the gating profile and substantially affects the response to oscillatory stimuli. Unlike other multivalent ions, Cu^2+^ potentiated the voltage-induced gating and open-close channel transitions were attained at smaller potentials. These effects were more pronounced for the close–open transitions: during descending voltage ramps, the channels did not reopen even at 0 mV bias potential (such measurements were made possible by using a new experimental setup), and large hyperpolarizing voltages were needed to reinstate the open channel conductance. The history-dependent transitions between conformations led to bistability, caused a strong hysteresis in conductance, and endowed the channels with potential memory capabilities. An interesting fact is that this molecular memory can be fully addressed by employing electrical signals for writing, reading, and erasing it. Lysenin channels behave like true biological memristors but the molecular mechanisms by which such intricate functions are attained are yet to be deciphered.

## Data Availability

The data presented in this study are available on request from the corresponding author.
